# Polypharmacy, chronic kidney disease, and mortality among older adults: A prospective study of National Health and nutrition examination survey, 1999–2018

**DOI:** 10.3389/fpubh.2023.1116583

**Published:** 2023-03-23

**Authors:** Xiaowen Wang, Chao Yang, Jie Jiang, Yonghua Hu, Yuantao Hao, Jia-Yi Dong

**Affiliations:** ^1^Peking University Center for Public Health and Epidemic Preparedness and Response, Peking University, Beijing, China; ^2^Public Health, Department of Social Medicine, Osaka University Graduate School of Medicine, Osaka, Japan; ^3^Renal Division, Department of Medicine, Peking University First Hospital, Peking University Institute of Nephrology, Beijing, China; ^4^Department of Epidemiology and Biostatistics, School of Public Health, Peking University Health Science Center, Beijing, China

**Keywords:** NHANES, chronic kidney disease, mortality, elderly, cohort, polypharmacy

## Abstract

**Introduction:**

Polypharmacy might contribute to a range of adverse outcomes, which could get worse in the elderly with chronic kidney disease (CKD). Evidence on polypharmacy, CKD, and mortality is scarce. We aimed to investigate the prospective association between polypharmacy, CKD and all-cause and cause-specific mortality in adults aged ≥65 years.

**Methods:**

A total of 13,513 adults from the National Health and Nutrition Examination Surveys were included, following up from 1999 to 2018 until December 31, 2019. The simultaneous use of ≥5 medications by one individual was defined as polypharmacy. Survey-weighted Cox proportional hazard models were used to estimate the hazard ratio (HRs) for mortality from all-cause, cardiovascular diseases (CVD), and cancer after adjusting for potential confounding factors.

**Results:**

Among the elderly with CKD, we identified 3,825 total deaths (1,325 CVD and 714 cancer) during a median follow-up of 7.7 years. Participants with polypharmacy had a 27% (HR = 1.27 [1.15, 1.39]) and 39% (HR = 1.39 [1.19, 1.62]) higher risk of all-cause and CVD mortality, respectively, but not for cancer mortality. Compared with the elderly with no polypharmacy and no CKD, the corresponding HRs (95%CIs) for all-cause mortality were 1.04 (0.96, 1.14) for those with no polypharmacy but CKD, 1.24 (1.11, 1.39) for with polypharmacy but no CKD, and 1.34 (1.21, 1.49) for those with both polypharmacy and CKD. A similar pattern was detected for CVD mortality.

**Discussion:**

Polypharmacy was associated with elevated risks of all-cause and CVD mortality among the elderly CKD patients. More evidence-based approaches should be promoted for the appropriate deprescribing in the older adults with CKD.

## 1. Introduction

Polypharmacy has emerged as an important public health issue in the population, especially among the elderly. There is no consensus as to the number of medications that amounts to polypharmacy, generally, it refers to the simultaneous use of five or more medications by one individual (1). The majority of old people suffered from multimorbidity, contributing to the high utilization of multiple medications or therapy. It was estimated that the prevalence of polypharmacy ranged from 27.0 to 96.5% in the older population in different healthcare settings (2, 3). Polypharmacy might contribute to a range of unfavorable effects and adverse outcomes in older adults, such as adverse drug events, hospitalizations, falls, impaired function, and cognition decline (4, 5). Mortality is one of the common and important measures used for epidemiological surveillance and indicates the progression and severity of a given health event (6). CVD and cancer are among the leading causes of mortality in the U.S. and are responsible for nearly half of all deaths (7). A meta-analysis showed a dose–response relationship between polypharmacy and mortality (8), and another recent meta-analysis revealed that polypharmacy was associated with a 28.0% higher risk of mortality in the elderly (≥65 years) (9). Furthermore, it was indicated by an English aging cohort that polypharmacy had a 2.45 times higher risk of mortality from cardiovascular disease (CVD) in people aged over 50, but cancer mortality was only related to heightened polypharmacy (taking over 10 medications) (10).

Chronic kidney disease (CKD) is a condition characterized by decline in kidney function. The prevalence of CVD and metabolic disease is notably higher among people with CKD, making them vulnerable to multimorbidity and polypharmacy (11–14). Previous research showed the prevalence of polypharmacy ranged from 62.0 to 86.0% in CKD patients (11, 15). Furthermore, the pharmacokinetics and pharmacodynamics might be altered due to kidney dysfunction in the elderly (16, 17), therefore the drug–drug interactions and drug adverse events might be amplificated in the aging CKD population (16, 18, 19). Currently, evidence on polypharmacy and health outcomes in older adults with CKD is scarce. Several cohort studies indicated polypharmacy was associated with an increased risk of kidney failure, fragility fracture, CVD events, and all-cause mortality in patients with CKD (20–22), even regardless of CKD status (20, 22, 23).

In this study, we aimed to investigate the association between polypharmacy, CKD and all-cause mortality, as well as CVD and cancer mortality in adults aged 65 years and old.

## 2. Methods

### 2.1. Study population

Data were extracted from the National Health and Nutrition Examination Surveys (NHANES), a nationally representative survey designed to evaluate the health and nutritional status of community-dwelling U.S. adults. NHANES repeats the cycle of surveys every 2 years with a methodology of multistage probability sampling design. The surveys combined self-administrated interviews with physical examinations and laboratory tests. Data were available at.^1^ NHANES has got approval from the Institutional Review Board of the National Center for Health Statistics. All participants have signed informed consent forms.

**Figure 1:**
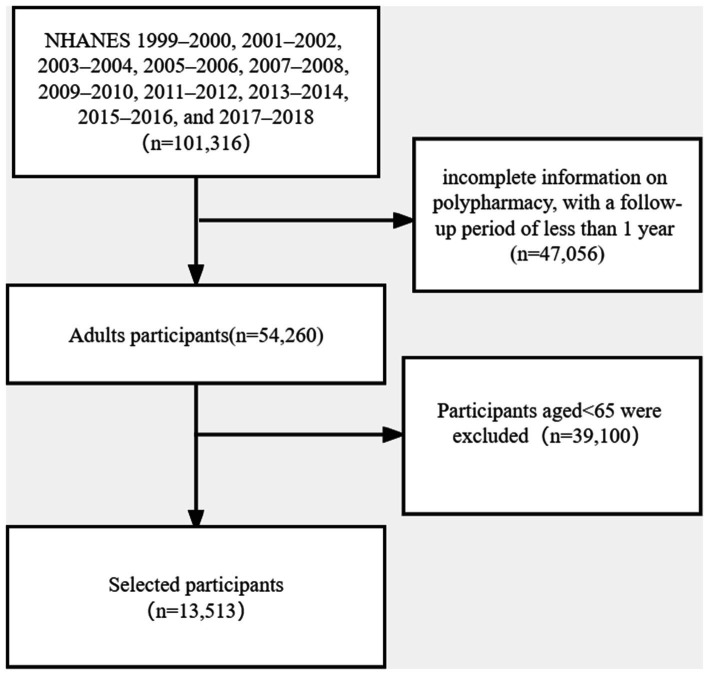
Flow chart of the participants selection.

In this present study, a total of 101,316 individuals from ten cycles (1999–2000, 2001–2002, 2003–2004, 2005–2006, 2007–2008, 2009–2010, 2011–2012, 2013–2014, 2015–2016, and 2017–2018) was included. Mortality follow-up data were recorded until the date of 31 December 2019. We excluded those younger than 65 years, those with incomplete information on polypharmacy, and with a follow-up period of less than 1 year. Finally, 13,513 individuals were included in the analysis (Figure 1).

### 2.2. Assessment of polypharmacy

During the in-home interview, participants would be asked, “Have you taken any prescription medications in the past month?” and those who answered “yes” would be further asked to show the containers of all medications they were using. At the same time, the interviewer would record the number and name of the participant’s prescription medications. Simultaneous use of five or more medications was defined as “polypharmacy.”

### 2.3. Assessment of chronic kidney disease

We calculated the glomerular filtration rate (GFR) estimated according to the Chronic Kidney Disease-Epidemiology Collaboration (CKD-EPI) equation (24): (1) for women, if serum creatinine ≤0.7 mg/dl, GFR = 144 × (serum creatinine (mg/dl)/0.7)^−0.329^ × (0.993)^age^; if serum creatinine >0.7 mg/dl, GFR = 144 × (serum creatinine (mg/dl)/0.7)^−1.209^ × (0.993)^age^. (2) For men, if serum creatinine ≤0.9 mg/dl, GFR = 141 × (serum creatinine (mg/dl)/0.7)^−0.441^ × (0.993)^age^; if serum creatinine >0.9 mg/dl, GFR = 144 × (serum creatinine (mg/dl)/0.7)^−0.441^ × (0.993)^age^. The serum creatinine measurements could be obtained from the ten cycles and recommended calibrations in NHANES had been adopted. We defined reduced estimated GFR (eGFR) as <60 ml/min/1.73 m^2^ and severely reduced eGFR as <30 ml/min/1.73 m^2^. CKD was defined as reduced eGFR. Albuminuria, defined as a urine albumin-to-creatinine ratio ≥ 30 mg/g, was further added for CKD outcomes (25).

### 2.4. Covariates

The demographics and socioeconomics included sex, age, race (Mexican American, other Hispanic, Non-Hispanic White, Non-Hispanic Black), and other race - including Multi-Racial, education (high school or below, and college or above), and family income-to-poverty ratio (≤1.0, and > 1.0). Body mass index (BMI) was calculated as weight/(height*height) (kg/m^2^) (underweight: BMI < 18.5; normal weight: 18.5 ≤ BMI < 25; overweight: 25 ≤ BMI < 30; obesity: BMI ≥ 30). Drinking status was based on the question “Had at least 12 alcohol drinks/1 year?,” with the answer of “yes” or “no.” The medical history included diabetes, hypertension, high cholesterol, heart disease (congestive heart failure, coronary heart disease, angina/angina pectoris, or heart attack), respiratory disease (asthma, emphysema, or chronic bronchitis), and cancer.

### 2.5. Ascertainment of death

The deidentified and anonymized participant data in NHANES were linked to longitudinal Medicare and mortality data from National Death Index (NDI) until the date of 31 December 2019. The cause of death was based on the International Classification of Diseases, 10th edition (ICD-10). Mortality follow-up data were recorded for participants from the start of the study until to date of death or 31 December 2019, whichever came first. The all-cause mortality and mortality from cardiovascular diseases (I00-I09, I11, I13, I20-I51), and cancer (C00-C97) were evaluated.

### 2.6. Statistical analysis

The characteristics of the included participants at baseline were presented according to polypharmacy. Continuous variables were shown as mean ± standard deviation. Categorical variables were expressed as numbers (%). Survey-weighted regression models were used to calculate the rate of polypharmacy among participants with CKD. Survey-weighted multivariable Cox proportional hazard models were used to calculate the hazard ratios (HRs) with 95% confidence intervals (CIs) of mortality by polypharmacy among all the participants as well as participants with CKD. The proportional hazards assumption tests were conducted by comparing models with and without a multiplicative interaction term between polypharmacy and follow-up duration. We detected no evidence of assumption violations in every model (*p* > 0.05 for all tests). Model 1 was adjusted for sex and age. Model 2 was further adjusted for race, education, income, BMI, drinking status, diabetes, hypertension, high cholesterol, heart disease, respiratory disease, and cancer. Missing values were treated as separate categories. Furthermore, the joint effect of polypharmacy and CKD on mortality among the older adults were analyzed. We also did sensitivity analyses by dividing polypharmacy as minor polypharmacy (5–9 medications) and major polypharmacy (≥10 medications), to detect the association between polypharmacy and mortality. SAS version 9.4 TS Level 1 M6 software (SAS Institute Inc., Cary, NC, United Sates) was used for all statistical analyses.

## 3. Results

Table 1 shows the characteristics of the included participants aged 65 or older at baseline according to polypharmacy. Older adults with polypharmacy were more likely to be slightly older, to be women, to be non-Hispanic White, and to have obesity, hypertension, diabetes, high cholesterol, heart disease, respiratory disease, and cancer., and a lower level of eGFR. A similar pattern was observed among older adults with CKD.

**Table 1:** Characteristics of adults aged 65 or older at baseline according to polypharmacy.

	All participants		Participants with CKD	
	No polypharmacy (*n* = 9,265)	Polypharmacy (*n* = 4,248)	No polypharmacy (*n* = 4,759)	Polypharmacy (*n* = 2,362)
**Sex**				
Men	4,597 (49.6)	1982 (46.7)	3,392 (71.3)	1,575 (66.7)
Women	4,668 (50.4)	2,266 (53.3)	1,367 (28.7)	787 (33.3)
Age, year	73.9 ± 6.0	74.7 ± 5.7	74.9 ± 6.0	75.5 ± 5.6
**Race**				
Mexican American	1,273 (13.7)	440 (10.4)	516 (10.8)	186 (7.9)
Other Hispanic	660 (7.1)	247 (5.8)	277 (5.8)	109 (4.6)
Non-Hispanic White	5,158 (55.7)	2,551 (60.1)	2,747 (57.7)	1,456 (61.6)
Non-Hispanic Black	1,583 (17.1)	771 (18.1)	963 (20.2)	490 (20.7)
Other Race - Including Multi-Racial	591 (6.4)	239 (5.6)	256 (5.4)	121 (5.1)
**Education**				
High school or below	5,545 (59.8)	2,520 (59.3)	2,866 (60.2)	1,382 (58.5)
College or above	3,665 (39.6)	1716 (40.4)	1851 (38.9)	972 (41.2)
**Family income-to-poverty ratio**				
≤1.0	2,568 (27.7)	1,106 (26.0)	1,296 (27.2)	605 (25.6)
>1.0	6,697 (72.3)	3,142 (74.0)	3,463 (72.8)	1757 (74.4)
**BMI**				
Underweight	1,075 (11.6)	507 (11.9)	911 (19.1)	424 (18.0)
Normal weight	2,454 (26.5)	787 (18.5)	1,136 (23.9)	383 (16.2)
Overweight	3,221 (34.8)	1,379 (32.5)	1,594 (33.5)	755 (32.0)
Obesity	2,515 (27.1)	1,575 (37.1)	1,118 (23.5)	800 (33.9)
**Drinking**				
Non-drinker	2,738 (29.6)	1,269 (29.9)	1,070 (22.5)	580 (24.6)
Drinker	4,441 (47.9)	1963 (46.2)	2,329 (48.9)	1,124 (47.6)
Diabetes	1,451 (15.7)	1,600 (37.7)	819 (17.2)	924 (39.1)
Hypertension	4,924 (53.1)	4,341 (82.0)	2,629 (55.2)	1829 (77.4)
High cholesterol	4,052 (43.7)	2,565 (60.4)	1964 (41.3)	1,360 (57.6)
Heart disease	1,513 (16.3)	1,488 (35.0)	997 (20.9)	974 (41.2)
Respiratory disease	1,363 (14.7)	1,027 (24.2)	676 (14.2)	517 (21.9)
Cancer	1944 (21.0)	1,100 (25.9)	1,111 (23.3)	667 (28.2)
eGFR, mL/min/1.73m^2^	68.3 ± 21.4	63.7 ± 22.6	46.8 ± 10.3	43.6 ± 11.9

Data are mean ± standard deviation or number (%). CKD, chronic kidney disease; BMI, body mass index; eGFR, estimated glomerular filtration rate.

Among all the elderly participants, we identified 6,049 total deaths, including 2067 CVD deaths and 1,134 cancer deaths during a median follow-up of 7.9 years. As shown in Table 2, after adjusting for age and sex (model 1), compared with the no polypharmacy group, the elderly with polypharmacy had a 46% (HR, 95%CI = 1.46 [1.36, 1.56]) and 77% (HR, 95%CI = 1.77 [1.58, 1.99]) increase in the risk of all-cause mortality and CVD mortality (i.e., 1.46 times and 1.77 times more likely to experience all-cause death and CVD death), respectively. After further adjustment for race, education, income, BMI, drinking status, diabetes, hypertension, high cholesterol, heart disease, respiratory disease, and cancer (model 2), it showed a 27% (HR = 1.27 [1.17, 1.37]) and 39% (HR = 1.39 [1.22, 1.58]) higher risk of mortality from all-cause and CVD, respectively, among the elderly with polypharmacy, in comparison to those with no polypharmacy. However, the association was non-significant for cancer mortality in the two models (HR = 1.14 [0.97, 1.33] and HR = 1.07 [0.91, 1.27], respectively). Sensitivity analyses showed similar results of minor polypharmacy and major polypharmacy for mortality from all-cause, CVD, and cancer (Supplementary Table S1).

**Table 2:** Hazard ratios and 95% confidence intervals (HRs, 95% CIs) for mortality from cardiovascular disease, cancer, and all-cause according to polypharmacy among older adults.

	No polypharmacy (*n* = 9,265)	Polypharmacy (*n* = 4,248)
**All-cause**		
Number of events	3,986	2063
Person-years	77,155	29,514
Model 1 HR (95% CI)	1.00	**1.46 (1.36, 1.56)**
Model 2 HR (95% CI)	1.00	**1.27 (1.17, 1.37)**
**Cardiovascular disease**		
Number of events	1,272	795
Person-years	77,155	29,514
Model 1 HR (95% CI)	1.00	**1.77 (1.58, 1.99)**
Model 2 HR (95% CI)	1.00	**1.39 (1.22, 1.58)**
**Cancer**		
Number of events	793	341
Person-years	77,155	29,514
Model 1 HR (95% CI)	1.00	1.14 (0.97, 1.33)
Model 2 HR (95% CI)	1.00	1.07 (0.91, 1.27)

Model 1: adjusted for sex and age; Model 2: further adjusted for race, education, income, BMI, drinking status, diabetes, hypertension, high cholesterol, heart disease, respiratory disease, and cancer. Statistically significant values of *p*<0.05 will be formatted in bold.

In Table 3, among the elderly with reduced eGFR, we identified 3,825 total deaths, including 1,325 CVD deaths and 714 cancer deaths during a median follow-up of 7.7 years. In model 1, compared with the no polypharmacy group, participants with polypharmacy had 44% (HR = 1.44 [1.32, 1.57]), 77% (HR = 1.77 [1.53, 2.03]), and 21% (HR = 1.21 [1.01, 1.45]) higher risk of mortality from all-cause, CVD, and cancer, respectively. In model 2, the associations were still significant for all-cause and CVD mortality, the elderly with polypharmacy had a 27% (HR = 1.27 [1.15, 1.39]) and 39% (HR = 1.39 [1.19, 1.62]) higher risk of all-cause and CVD mortality, respectively. However, the association was not significant for cancer mortality (HR = 1.18 [0.98, 1.43]). Sensitivity analyses showed similar patterns for mortality from cardiovascular disease, cancer, and all-cause according to polypharmacy (Supplementary Table S2). Among the elderly with severely reduced eGFR (eGFR <30 ml/min/1.73m^2^), similar patterns of associations of polypharmacy with mortality from all-cause, CVD, and cancer were observed. After further adjustment for covariates, in comparison to those with no polypharmacy, the elderly with polypharmacy had a 24% (HR = 1.24 [1.07, 1.43]) and 54% (HR = 1.54 [1.21, 1.95]) higher risk of all-cause and CVD mortality, respectively, but not for cancer mortality (HR = 0.91 [0.68, 1.23]). Among the elderly with albuminuria, the association between polypharmacy and mortality showed similar results (Table 3). In the multivariable models, compared with no polypharmacy group, the elderly with polypharmacy had a 31% (HR = 1.31 [1.14, 1.50]) and 49% (HR = 1.49 [1.18, 1.90]) higher risk of all-cause and CVD mortality, respectively.

**Table 3:** Hazard ratios and 95% confidence intervals (HRs, 95% CIs) for mortality from cardiovascular disease, cancer, and all-cause according to polypharmacy and kidney function among participants with chronic kidney disease.

	No polypharmacy	Polypharmacy
eGFR < 60 ml/min/1.73m^2^	*n* = 4,759	*n* = 2,362
**All-cause**		
Number of events	2,483	1,342
Person-years	39,115	15,690
Model 1 HR (95% CI)	1.00	**1.44 (1.32, 1.57)**
Model 2 HR (95% CI)	1.00	**1.27 (1.15, 1.39)**
**Cardiovascular disease**		
Number of events	807	518
Person-years	39,115	15,690
Model 1 HR (95% CI)	1.00	**1.77 (1.53, 2.03)**
Model 2 HR (95% CI)	1.00	**1.39 (1.19, 1.62)**
**Cancer**		
Number of events	488	226
Person-years	39,115	15,690
Model 1 HR (95% CI)	1.00	1.21 (1.01, 1.45)
Model 2 HR (95% CI)	1.00	1.18 (0.98, 1.43)
**eGFR < 30 ml/min/1.73m** ^**2**^	***n* = 1906**	***n* = 900**
**All-cause**		
Number of events	1,130	565
Person-years	16,275	6,032
Model 1 HR (95% CI)	1.00	**1.47 (1.30, 1.67)**
Model 2 HR (95% CI)	1.00	**1.24 (1.07, 1.43)**
**Cardiovascular disease**		
Number of events	344	219
Person-years	16,275	6,032
Model 1 HR (95% CI)	1.00	**2.02 (1.64, 2.50)**
Model 2 HR (95% CI)	1.00	**1.54 (1.21, 1.95)**
**Cancer**		
Number of events	209	87
Person-years	16,275	6,032
Model 1 HR (95% CI)	1.00	1.05 (0.80, 1.39)
Model 2 HR (95% CI)	1.00	0.91 (0.68, 1.23)
**With albuminuria**	***n* = 1739**	***n* = 1,114**
**All-cause**		
Number of events	982	656
Person-years	12,470	6,629
Model 1 HR (95% CI)	1.00	**1.44 (1.28, 1.63)**
Model 2 HR (95% CI)	1.00	**1.31 (1.14, 1.50)**
**Cardiovascular disease**		
Number of events	340	261
Person-years	12,470	6,629
Model 1 HR (95% CI)	1.00	**1.70 (1.37, 2.11)**
Model 2 HR (95% CI)	1.00	**1.49 (1.18, 1.90)**
**Cancer**		
Number of events	166	99
Person-years	12,470	6,629
Model 1 HR (95% CI)	1.00	1.17 (0.88, 1.55)
Model 2 HR (95% CI)	1.00	1.15 (0.82, 1.61)

Model 1: adjusted for sex and age; Model 2: further adjusted for race, education, income, BMI, drinking status, diabetes, hypertension, high cholesterol, heart disease, respiratory disease, and cancer. Albuminuria was defined as urine albumin-to-creatinine ratio ≥ 30 mg/g; eGFR, estimated glomerular filtration rate. Statistically significant values of *p*<0.05 will be formatted in bold.

**Figure 2:**
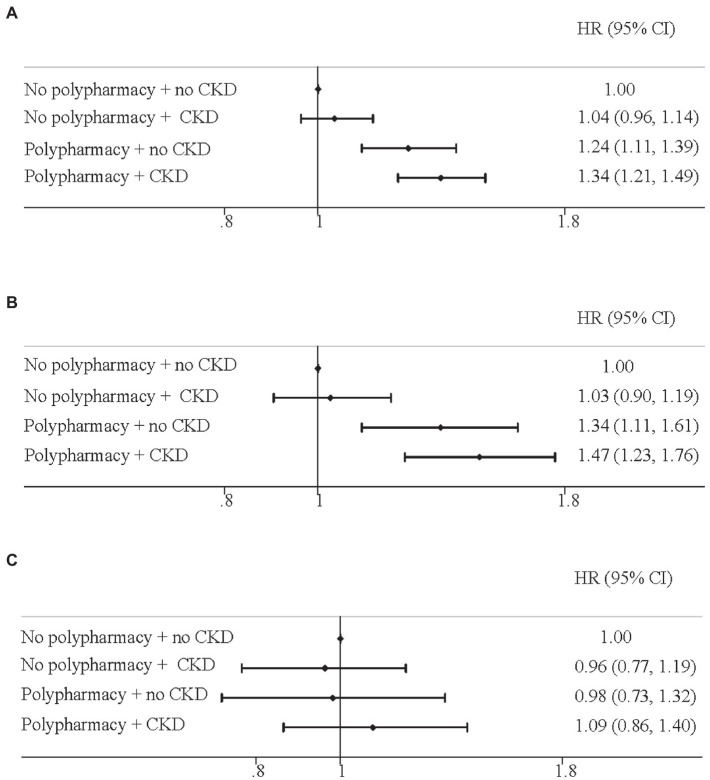
Joint effect of polypharmacy and chronic kidney disease on mortality for **(A)** all-cause; **(B)** cardiovascular disease; **(C)** cancer. Hazard ratios and 95% confidence intervals (HRs, 95% CIs) were adjusted for age, sex. Race, education, income, body mass index, drinking status, diabetes, hypertension, high cholesterol, heart disease, respiratory disease, and cancer.

We then conducted the joint-association analyses of polypharmacy and CKD on mortality from all-cause, CVD, and cancer in the elderly (Figure 2). For all-cause mortality, compared with the elderly with no polypharmacy and no CKD, the corresponding HRs (95%CIs) were 1.04 (0.96, 1.14) for those with no polypharmacy but CKD, 1.24 (1.11, 1.39) for with polypharmacy but no CKD, and 1.34 (1.21, 1.49) for those with both polypharmacy and CKD (Figure 2A). For CVD mortality, a similar pattern was detected where the corresponding HRs (95%CIs) were 1.03 (0.90, 1.19), 1.34 (1.11, 1.61), and 1.47 (1.23, 1.76), respectively (Figure 2B). However, we did not observe any significant association with cancer mortality (Figure 2C).

## 4. Discussion

In this prospective study, among the elderly with CKD, in comparison to those with no polypharmacy, individuals with polypharmacy had 1.27 times and 1.39 times the risk of all-cause and CVD mortality, respectively, but not for cancer mortality. The older adults with polypharmacy and CKD had the highest risk of mortality. A previous cohort comprised of U.S. adults aged ≥45 years with a 5-year follow-up showed that only major polypharmacy (≥ 8 medications) was associated with increased mortality (HR = 1.24 [1.06, 1.45]), but not for minor polypharmacy (6–7 medications) (HR = 1.15 [0.98, 1.36]) in those with CKD, and there was no evidence that this association was modified by CKD status (22). Another Japanese cohort consisting of 1,117 participants reported that both polypharmacy (5–9 medications) and hyperpolypharmacy (≥10 medications) were associated with kidney failure (HR = 2.28 [1.00, 5.21] and HR = 2.83 [1.21, 6.66], respectively), but only hyperpolypharmacy was related to all-cause mortality (HR = 2.80 [1.41–5.54]) (21). However, our sensitivity analysis suggested even minor polypharmacy significantly increased mortality in the elderly CKD patients. A review concluded that medication deprescribing interventions could offer small reductions in mortality (OR = 0.74 [0.58, 0.95]), though no significant effect on hospitalizations, falls, or health-related quality of life among community-dwelling people aged ≥65 years (26).

To our knowledge, we firstly reported the adverse impacts of polypharmacy on cardiovascular mortality in the older adults with CKD. People with CKD could release a high level of cytokines, hormones, and enzymes, as well as alter hemodynamics due to kidney insufficiency or injury (27–29). An international prescribing pattern survey showed approximately 39% of the participants most frequently prescribed cardiovascular medications and 91% were experiencing polypharmacy (13). It is controversial that polypharmacy was shown to be efficacious or safe (30–32). Polypharmacy might exert an additive effect on the cardiac damage caused by CKD, thus leading to the deterioration of CVD outcomes (31, 33, 34).

The highest mortality was observed in the elderly with both polypharmacy and CKD. The amount of medication taken may reflect the number of medical conditions they have and severity of the diseases. CKD patients who had comorbidities (e.g., diabetes, atrial fibrillation, hypertension) had increased mortality rates (35). Furthermore, it has been reported that polypharmacy is associated with a broad range of adverse outcomes, including bleeding, hospitalizations, falls, fractures, frailty, and poor health-related quality of life (4, 36). Also, polypharmacy might be associated with kidney impairment (37, 38). For example, a Switzerland study reported every additional prescribed drug was related to a decrease in eGFR by 0.39 ml/min per 1.73 m^2^ over 24 months (38). Kidney dysfunction could further exacerbate multimorbidity, hence higher levels of comorbidities and chronic inflammation could result in worse clinical consequences (34, 39–41). Interestingly, a study found while people with CKD had higher absolute risk of death, there was no difference in the relative risk associated with polypharmacy by CKD status. Polypharmacy-related adverse events could be mitigated if drug management was undertaken by a nephrologist in patients with much advanced CKD (23). Overall, our study indicated the importance of recommendations or guidelines for lowering medication burden to achieve appropriate and safe medication management in the elderly with CKD. So far, several trials have been attempted to improve the health outcomes intended for older patients receiving polypharmacy. For example, intervention on clinical geriatric assessment combined with a thorough medications review, as well as a meeting between the geriatrician and the family physician could contribute to positive impacts on health-related quality of life in home-dwelling older patients receiving polypharmacy (42, 43).

The present study was strengthened by using nationally representative population and a prospective cohort design. Several limitations should be addressed. First, we assessed the CKD by using serum creatinine according to a single measurement, which could lead to the misclassification of CKD. However, the misclassification was inclined to be non-differential. Second, we quantified the prescription by using the term of polypharmacy to indicate the overall medication burden by one individual, but did not further analyze the specific type of medication. Third, the outcomes included all-cause mortality and mortality from CVD and cancer, while other causes, such as sepsis, cerebrovascular diseases, influenza and pneumonia were not considered. Although CVD and cancer are among the leading causes of mortality in the U.S. and are responsible for approximately half of all deaths (7). Furthermore, the covariates for diseases, such as diabetes and hypertension were self-reported, though previous studies have supported the high validity of clinical records in NHANES (44–46). Lastly, it is possible that residual confounding existed due to some unmeasured variables (e.g., dietary factors).

## 5. Conclusions and implications

Polypharmacy might contribute to a range of adverse outcomes, which could get worse in the elderly with CKD. Since evidence on polypharmacy, CKD, and mortality was scarce, our study highlighted that polypharmacy was associated with higher risks of all-cause and CVD mortality among the older adults with CKD. The older adults with both polypharmacy and CKD had the highest risk of mortality. Our study also indicated the importance of recommendations or guidelines for lowering medication burden to achieve appropriate and safe medication management in the elderly with CKD. More evidence-based approaches, such as clinical geriatric assessment and collaborative medication reviews by physicians should be promoted for the appropriate deprescribing in the older adults with CKD.

## Data availability statement

The datasets presented in this study can be found in online repositories. The names of the repository/repositories and accession number(s) can be found at: https://www.cdc.gov/nchs/nhanes/index.htm.

## Ethics statement

The studies involving human participants were reviewed and approved by NHANES has got approval from the Institutional Review Board of the National Center for Health Statistics. The patients/participants provided their written informed consent to participate in this study.

## Author contributions

XW: conceptualization, formal analysis, data curation, and writing−original draft preparation. J−YD: conceptualization, formal analysis, and writing−reviewing and editing. CY: data curation and writing−reviewing and editing. JJ: writing−reviewing and editing. YH: validation, and writing−reviewing and editing. YH: supervision, validation, and writing−reviewing and editing. All authors contributed to the article and approved the submitted version.

## Funding

This work is supported by the National key research and development program of China (grant no. 2022YFC3600804).

## Conflict of interest

The authors declare that the research was conducted in the absence of any commercial or financial relationships that could be construed as a potential conflict of interest.

## Publisher’s note

All claims expressed in this article are solely those of the authors and do not necessarily represent those of their affiliated organizations, or those of the publisher, the editors and the reviewers. Any product that may be evaluated in this article, or claim that may be made by its manufacturer, is not guaranteed or endorsed by the publisher.

## Footnotes

^1^ https://www.cdc.gov/nchs/nhanes/index.htm
