# Supplementary material for: Polypharmacy, chronic kidney disease, and mortality among older adults: A prospective study of National Health and nutrition examination survey, 1999–2018
Source: Front Public Health. 2023 Mar 23;11:1116583. doi: 10.3389/fpubh.2023.1116583 (PMC10077868; doi:10.3389/fpubh.2023.1116583)
Supplement: Supplementary file 1 [file Table_1.DOCX]

Supplementary Table 1. Hazard ratios and 95 % confidence intervals (HRs, 95 % CIs) for mortality from cardiovascular disease, cancer, and all-cause according to polypharmacy.

|  | No polypharmacy (<5 medication) (n=9265) | Minor Polypharmacy (5-9 medication) (n=4189) | Major Polypharmacy (≥10 medication) (n=59) |
| --- | --- | --- | --- |
| **All-cause** |  |  |  |
| Number of events | 3986 | 2008 | 55 |
| Person-years | 77,155 | 29,046 | 468 |
| Model 1 HR (95 % CI) | 1.00 | 1.44 (1.34, 1.54) | 2.51 (1.84, 3.42) |
| Model 2 HR (95 % CI) | 1.00 | 1.26 (1.16, 1.36) | 1.84 (1.37, 2.47) |
| **Cardiovascular disease** |  |  |  |
| Number of events | 1272 | 776 | 19 |
| Person-years | 77,155 | 29,046 | 468 |
| Model 1 HR (95 % CI) | 1.00 | 1.75 (1.56, 1.96) | 3.44 (1.94, 6.08) |
| Model 2 HR (95 % CI) | 1.00 | 1.38 (1.22, 1.57) | 2.05 (1.16, 3.63) |
| **Cancer** |  |  |  |
| Number of events | 793 | 332 | 9 |
| Person-years | 77,155 | 29,046 | 468 |
| Model 1 HR (95 % CI) | 1.00 | 1.13 (0.96, 1.33) | 1.75 (0.92, 3.30) |
| Model 2 HR (95 % CI) | 1.00 | 1.07 (0.90, 1.26) | 1.51 (0.76, 3.00) |

Model 1: adjusted for sex and age;

Model 2: further adjusted for race, education, income, BMI, drinking status, diabetes, hypertension, high cholesterol, heart disease, respiratory disease, and cancer.

Supplementary Table 2. Hazard ratios and 95 % confidence intervals (HRs, 95 % CIs) for mortality from cardiovascular disease, cancer, and all-cause according to polypharmacy among participants with chronic kidney disease (eGFR <60 mL/min/1.73m^2^).

|  | No polypharmacy (<5 medication) (n=4759) | Minor Polypharmacy (5-9 medication) (n=2319) | Major Polypharmacy (≥10 medication) (n=43) |
| --- | --- | --- | --- |
| All-cause |  |  |  |
| Number of events | 2483 | 1301 | 41 |
| Person-years | 39,115 | 15,350 | 340 |
| Model 1 HR (95 % CI) | 1.00 | 1.55 (1.42, 1.68) | 2.58 (1.94, 3.42) |
| Model 2 HR (95 % CI) | 1.00 | 1.28 (1.16, 1.41) | 1.82 (1.36, 2.43) |
| Cardiovascular disease |  |  |  |
| Number of events | 807 | 503 | 15 |
| Person-years | 39,115 | 15,350 | 340 |
| Model 1 HR (95 % CI) | 1.00 | 1.92 (1.68, 2.20) | 4.00 (2.41, 6.66) |
| Model 2 HR (95 % CI) | 1.00 | 1.44 (1.24, 1.67) | 2.29 (1.39, 3.76) |
| Cancer |  |  |  |
| Number of events | 488 | 220 | 6 |
| Person-years | 39,115 | 15,350 | 340 |
| Model 1 HR (95 % CI) | 1.00 | 1.26 (1.06, 1.50) | 1.61 (0.73, 3.52) |
| Model 2 HR (95 % CI) | 1.00 | 1.15 (0.94, 1.41) | 1.34 (0.58, 3.09) |

Model 1: adjusted for sex and age;

Model 2: further adjusted for race, education, income, BMI, drinking status, diabetes, hypertension, high cholesterol, heart disease, respiratory disease, and cancer.

eGFR, estimated glomerular filtration rate.
